# Estimation of global and local complexities of brain networks: A random walks approach

**DOI:** 10.1162/netn_a_00138

**Published:** 2020-07-01

**Authors:** Roberto C. Sotero, Lazaro M. Sanchez-Rodriguez, Narges Moradi, Mehdy Dousty

**Affiliations:** Hotchkiss Brain Institute, University of Calgary, AB, Canada; Department of Radiology, University of Calgary, AB, Canada; Biomedical Engineering Graduate Program, University of Calgary, AB, Canada; Hotchkiss Brain Institute, University of Calgary, AB, Canada; Department of Radiology, University of Calgary, AB, Canada; Hotchkiss Brain Institute, University of Calgary, AB, Canada; Department of Radiology, University of Calgary, AB, Canada; Biomedical Engineering Graduate Program, University of Calgary, AB, Canada; Institute of Biomaterials and Biomedical Engineering, University of Toronto, ON, Canada; KITE, Toronto Rehab, University Health Network, Toronto, ON, Canada

**Keywords:** Network complexity, Local complexity, Random walk, Sample entropy, Resting-state networks

## Abstract

The complexity of brain activity has been observed at many spatial scales and has been proposed to differentiate between mental states and disorders. Here we introduced a new measure of (global) network complexity, constructed as the sum of the complexities of its nodes (i.e., local complexity). The complexity of each node is obtained by comparing the sample entropy of the time series generated by the movement of a random walker on the network resulting from removing the node and its connections, with the sample entropy of the time series obtained from a regular lattice (ordered state) and a random network (disordered state). We studied the complexity of fMRI-based resting-state networks. We found that positively correlated (**pos**) networks comprising only the positive functional connections have higher complexity than anticorrelation (**neg**) networks (comprising the negative connections) and the network consisting of the absolute value of all connections (**abs**). We also observed a significant correlation between complexity and the strength of functional connectivity in the pos network. Our results suggest that the pos network is related to the information processing in the brain and that functional connectivity studies should analyze **pos** and **neg** networks separately instead of the **abs** network, as is commonly done.

## Author Summary

Current measures of network complexity fail to capture the structural and functional diversity of brain networks, in which hierarchies of linked communities span across several spatial scales, from cortical minicolumns to large-scale networks. In this paper we use random walks processes to obtain a time series reflecting the complex structure of brain networks and use this time series to construct measures of local and global complexity. We found that complexity is significantly correlated to the strength of the connections in the positively correlated brain network, being stronger at the global than at the local scale.

## INTRODUCTION

The development of a quantitative measure of complexity has proven difficult because of the variety of systems that may be labeled as “complex.” In the case of the complexity of networks, perhaps the most popular approach has been the use of information-based measures (Bonchev & Buck, 2005; Dehmer & Barbarini, 2009). The basic principle to construct these measures is to select an arbitrary graph invariant *X*, partitioned as *X*_1_, …, *X*_*N*_. Probabilities can be inferred for each partition using the entities *p*_*i*_ = *X*_*i*_/$\sum_{i=1}^{N}$*X*_*i*_ since it holds that $\sum_{i=1}^{N}$*p*_*i*_ = 1. The information content of the graph is then computed using the Shannon formula (Shannon, 1948): *H* = $\sum_{i=1}^{N}$*p*_*i*_*log*(*p*_*i*_). Another important definition of complexity was proposed by Kolmogorov (1968). The Kolmogorov complexity of a network is the length of the shortest computer program that produces the network as output. Although Kolmogorov complexity is uncomputable it can be approximated to a degree that allows its practical use (Li & Vitányi, 2008).

The measures of complexity described above assume it to be a monotonically increasing function of disorder. However, complexity can also be defined as a monotonically increasing function of order, as shown by McShea (1991), who found that the morphological complexity of organisms changed with the level of self-organization, and the latter with order. Finally, complexity can be defined as a convex function of disorder; that is, a quantity that attains a minimum for both completely ordered and completely disordered systems, and a maximum at some intermediate level of disorder or order (López-Ruiz, Mancini, & Calbet, 1995; Shiner, Davison, & Landsberg, 1999; Tononi, Edelman, & Sporns, 1998). Here, we adopt this latter notion by assuming that network complexity achieves a minimum for regular lattice (RL) networks (Watts & Strogatz, 1998) and random networks, also known as Erdös–Rényi (ER) networks (Erdös & Rényi, 1959).

In addition to the global complexity of the brain network, in this work we are interested in computing the local complexities (a measure for each of the different brain areas), such that the global complexity of the network is the sum of the local ones; that is, the complexity of the system is the sum of the complexity of its parts. To estimate the complexities, we let random walkers diffuse on the network and construct time series of the strengths of the nodes (brain areas) visited by each of the walkers. The sample entropy (SampEn) (Richman & Moorman, 2000) of the time series is then calculated. Local complexities are obtained by iteratively removing a node and all its connections, constructing the time series from the walker movement in the resulting network, computing the SampEn, and comparing this value to the average value obtained from 1,000 ER and 1,000 RL networks with the same degree distribution and connections strengths.

Functional connectivity in the brain is defined as the synchronization of neurophysiological events among anatomically separated brain areas (Friston, Jezzard, & Turner, 1994). Biswal, Yetkin, Haughton, and Hyde (1995) were the first to report that during resting state the primary motor regions in the left and right hemispheres were positively correlated. Later studies identified positive correlations between regions that are now known to comprise the default mode network (DMN) (Buckner, Andrews-Hanna, & Schacter, 2008; Raichle, Snyder, Powers, Gusnard, & Shulman, 2001). In addition to the reported correlated networks, anticorrelated networks have also been reported by several studies (Fox, Zhang, Snyder, & Raichle, 2009; Gopinath, Krishnamurthy, Cabanban, & Crosson, 2015; Liang, King, & Zhang, 2012). Although anticorrelations have been attributed to the global signal removal, recent studies suggest a physiological basis (Fox et al., 2009; Kazeminejad & Sotero, 2019). For this reason, in this paper we computed three different functional connectivity matrices for each subject by using the Pearson correlation between the resting-state functional magnetic resonance imaging (fMRI) signals recorded from each of the 116 brain areas considered. A matrix consisting of the absolute value of all connections (denoted as **abs**), a matrix consisting of only the positive connections (denoted as **pos**) representing the positively correlated network, and a matrix comprising the absolute value of only the negative connections (denoted as **neg**) representing the anticorrelation network. We then compute the local complexities of the 116 brain areas, as well as the global complexities of the entire brain network, and seven known functional networks of the brain (Sedeño et al., 2016): default mode network (DMN), fronto-parietal (FP), salience (SAL), sensorimotor (SM), visual (V), cerebellar (CER), and temporo-basal-ganglial (TBG) networks. Our results show that the **pos** network has higher global complexity than the **neg** and **abs** networks. We also found that the link between complexity and functional connectivity is stronger for the pos network than for **neg** network, and changes with the spatial scale for the **pos** network, being stronger at the global scale than at the local scale. Also, in the **pos** network global complexity was strongly correlated to the network integration and segregation, whereas neg and abs were not significantly correlated with integration and segregation. Our results suggest that the **pos** network is related to the information processing in the brain network and should be used for functional connectivity analysis instead of the abs network.

## METHODS

### Data Acquisition and Preprocessing

We requested and received access to data collected by NIH Human Connectome Project (HCP) for the purpose of scientific investigation and agreed to their open-access terms of use. The resting-state fMRI dataset of 89 subjects from the HCP (https://db.humanconnectome.org) (Van Essen et al., 2013) was used in this research. The HCP consent procedure was approved by the Washington University institutional review board. For more information see Van Essen et al. (2013). Each subject was involved in four runs of 15 minutes each using a 3 T Siemens scanner while their eyes were open and had a relaxed fixation on a projected bright cross-hair on a dark background. The data were acquired with 2.0-mm isotropic voxels for 72 slices, TR = 0.72 s, TE = 33.1 ms, 1,200 frames per run, 0.58-ms echo spacing, and 2,290 Hz/Px bandwidth (Moeller et al., 2010). Therefore, the fMRI data were acquired with a spatial resolution of 2 × 2 × 2 mm and a temporal resolution of 0.72 s, using multibands accelerated echo-planar imaging to generate a high quality and the most robust fMRI data. The fMRI data were spatially preprocessed to remove spatial artifacts produced by head motion, *B*_0_ distortions, and gradient nonlinearities (Jovicich et al., 2006). Since comparison of fMRI images across subjects and studies is possible when the images have been transformed from the subject’s native volume space to the Montreal Neurological Institute (MNI) space, fMRI images were wrapped and aligned into the MNI space with FSL’s FLIRT 12 DOF affine and then an FNIRT nonlinear registration (Jenkinson, Bannister, Brady, & Smith, 2002) was performed. In this study, the MNI-152-2mm atlas (Mazziotta et al., 2001) was utilized for fMRI data registration.

### Construction of Functional Connectivity Matrices

The peak voxel in each region, that is, the voxel of maximal activation, was selected by computing the root-mean-square for each voxel’s fMRI signal over all time. It has been shown that the peak voxel provides the best effect of any voxel in the region of interest (ROI) (Sharot, Delgado, & Phelps, 2004). Additionally, the peak voxel activity correlates better with evoked scalp electrical potentials than the average activity across the ROI. This means that the peak voxel represents the ROI’s activity better than other choices (Arthurs & Boniface, 2003). The peak voxel in each region is determined using previously published Talairach coordinates (after conversion to MNI coordinates and using Automated Anatomical Labeling (AAL) 116 atlas) (Fox et al., 2005). The resulting signal was filtered to keep only low-frequency fluctuations (0.01–0.08 Hz) (Yan & Zang, 2010). Finally, the global signal (i.e., the average of the fMRI signals over the whole brain (Moradi, Dousty, & Sotero, 2019)) was regressed out.

We then computed the Pearson correlation between all possible pairs of time series, creating a 116 × 116 functional connectivity matrix for each subject. In all cases *p* values were corrected by means of a multiple comparison analysis based on the false discovery rate (Benjamini & Hochberg, 1995). Three different networks were obtained from this matrix. A network consisting of the absolute value of all connections (denoted as **abs**) which is the most commonly used in fMRI connectivity studies (Meier et al., 2016; Meszlényi, Hermann, Buza, Gál,& Vidnyánszky, 2017; Salvador et al., 2005), a network consisting of only the positive connections (denoted as **pos**), and a network comprising the absolute value of only the negative connections (denoted as **neg**).

### Construction of Anatomical Connectivity Matrices

The HCP preprocessed diffusion data and the structural preprocessed data were used to compute the structural connectivity for each subject. The preprocessed steps in HCP were conducted by using FSL and Freesurfer softwares (Sotiropoulos et al., 2013). The following describes the preprocessing steps for the DTI data. Six diffusion series were used to normalize the intensity of mean b0 images. Several algorithms implemented in FSL were used to remove distortions, that is, the TOPUP algorithm to remove the echo planner distortion and the EDDY algorithm to correct the Eddy current-induced distortions and subject motion. The registration was done by using the FLIRT and FreeSurfer’s Bbregister algorithms (Sotiropoulos et al., 2013). The T1W images were parcellated with the IBASPM toolbox (Alemán-Gómez, Melie-Garcia, & Valdés-Hernández, 2006) into AAL 116. The MRtrix toolbox was used to perform diffusion-weighted MRI white matter tractography by using constrained spherical deconvolution and a probabilistic streamlines algorithm (Tournier, Calamante, & Connelly, 2012). A weighted structural connectivity matrix was obtained after eliminating volume and fiber length biases (Tournier et al., 2012).

### Construction of the Time Series of the Random Walker’s Movements on the Connectivity Matrix

We first consider an unweighted network consisting of *N* nodes. We place a large number *K* (*K* ≫ *N*) of random walkers onto this network. At each time step, the walkers move randomly (with the same probability) between the nodes that are directly linked to each other. We allow the walkers to perform *T* time steps. As a walker visits a node, we record the degree of the node. Thus, after *T* time steps, we obtain *K* time series reflecting different realizations of the random walker’s movement on the network. Nodes with high degree (hubs) will appear more frequently in the series than nodes with low degree.

In the case of weighted networks, such as the functional connectivity matrix representing the brain network, the routing strategy is a biased random walker, where the motion of a random walker located at a given node is biased according to the weights of the connections to the neighboring nodes (Zhang, Shan, & Chen, 2013). Specifically, the transition probability *p*_*ij*_ from brain area *i* to brain area *j* is given by *p*_*ij*_ = *w*_*ij*_/$\sum_{j=1}^{N}$*w*_*ij*_, where *w*_*ij*_ is the weight of the connection from area *i* to area *j* (Sotero, Sanchez-Rodriguez, Dousty, Iturria-Medina, & Sanchez-Bornot, 2019). We then construct a time series with the strengths of the nodes *i* visited by the walker: *str*_*i*_ = $\sum_{j=1}^{N}$*w*_*ij*_.

### Computing the Entropy of the Time Series

In this paper we use sample entropy (SampEn) (Richman & Moorman, 2000) to estimate the complexity of the time series of the diffusion of the random walker in the network. SampEn improved from approximate entropy (ApEn) (Pincus, 1991) by reducing the bias caused by self-matching. For a time series *x*(*i*), 1 ≤ *i* ≤ *N*, of finite length *N*, we first reconstitute the *N* − *m* + 1 vectors *X*_*m*_(*i*) following the form:

$$\begin{array}{c} X_{m}(i)=\left\{x(i),x(i+1),\ldots ,x\left(i+m-1\right)\right\},\\ i=1,2,\ldots ,N-m+1 \end{array}$$ (1)

where *m* is the embedding dimension, that is, the minimum dimension required to reconstruct the phase space of the time series (Cao, 1997). SampEn is the negative logarithm of the probability that if two sets of simultaneous data points of length *m* have distance smaller than the tolerance *r*, then two sets of simultaneous data points of length *m* + 1 also have distance smaller than *r*. Mathematically, we start by defining the probability $G_{i}^{m}$(*r*) that any vector *X*_*m*_(*j*) is within distance *r* of *X*_*m*_(*i*):

$$G_{i}^{m}(r)=\frac{1}{N-m+1}\sum_{j=1}^{N-m+1}\Theta \left(d_{ij}^{m}-r\right)$$ (2)

where $d_{ij}^{m}$ is the distance between the vectors *X*_*m*_(*i*) and *X*_*m*_(*j*), defined as:

$$\begin{array}{c} d_{ij}^{m}=max\left(\left|x(i+k)-x\left(j-k\right)\right|\right),\\ k=0,1,\ldots ,m \end{array}$$ (3)

When the embedding dimension is m, the total number of template matches is:

$$B_{m}(r)=\frac{1}{N-m}\sum_{i=1}^{N-m}G_{i}^{m}(r)$$ (4)

Similarly, when the embedding dimension is *m* + 1, the total number of template matches is:

$$A_{m}(r)=\frac{1}{N-m}\sum_{i=1}^{N-m}G_{i}^{m+1}(r)$$ (5)

Finally, the SampEn of the time series is estimated by:

$$SampEn(r,m,N)=-ln\left(\frac{A_{m}(r)}{B_{m}(r)}\right)$$ (6)

For all calculations, we take the value of m to be 2 and the value of r to be 0.2 std, where std is the standard deviation of the time series which should be taken over a large dataset (Delgado-Bonal & Marshak, 2019). Supporting Information Figure S1 shows the sample entropy of the time series constructed with the strengths of the nodes visited by a random walker released on an ER network (*N* = 100) for different values of the length of the time series. We see that for series comprising more than 23,000 points there is no significant change in the sample entropy when the length is increased. Based on this simulation, we chose a length of 25,000 points for all calculations of the entropy.

### Computing Local Complexities and Global Complexity

We propose to obtain local complexities *c*_*i*_ by (1) iteratively removing a node and all its connections, (2) constructing the time series from the random walker diffusion in the resulting network, and (3) computing the SampEn of the time series obtained in the previous step. For node *i*, the resulting SampEn is labeled as *H*_≠*i*_. Then we compare this entropy to the average SampEn (computed following the same procedure outlined before) of 1,000 ER (${\overset{-}{H}}_{ER}$) and 1,000 RL networks (${\overset{-}{H}}_{RL}$) of the same size (i.e, *N* − 1) and connections strengths taken from the original matrix. The local complexity is the percent this comparison is of the square of the entropy of the original matrix (*H*), multiplied by the probability (*p*_*i*_) of the appearance of the node in the time series:

$$c_{i}=100p_{i}\frac{\left|\left(H_{\neq i}-{\overset{-}{H}}_{ER}\right)\left(H_{\neq i}-{\overset{-}{H}}_{RL}\right)\right|}{H^{2}}$$ (7)

Note that the connection strengths of the 1,000 ER and 1,000 RL networks used to compute ${\overset{-}{H}}_{ER}$ and ${\overset{-}{H}}_{RL}$ in Equation 7 are generated using a probability density function estimated from the original matrix by means of a kernel density estimator (Bowman & Azzalini, 1997) as implemented by Matlab’s function “ksdensity.” In the case of the functional and anatomical brain connectivity data, we construct a vector with the nonzero strengths from all subjects and use as input to the ksdensity function.

**Figure 1.**
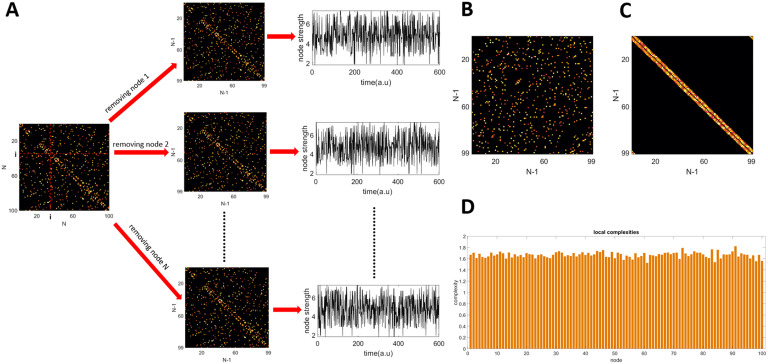
Methodology for computing local complexities. (A) Given a connectivity matrix of size N, each node is removed iteratively and a new matrix of size (*N* − 1) × (*N* − 1) is obtained. Then a time series of node strengths is constructed from the diffusion of a random walker in the new matrix. (B) A random network of size (*N* − 1) × (*N* − 1) with the same average degree and strengths as the matrices obtained in A. (C) A regular network of size (*N* − 1) × (*N* − 1) with the same average degree and strengths as the matrices obtained in A. (D) Local complexities.

Figure 1 shows the steps described above for computing the local complexities. The global complexity of the network *C* is then computed as the sum of the local complexities:

$$C=\sum_{i=1}^{N}c_{i}$$ (8)

### Measures of Network Integration and Segregation

Segregation and integration are two complementary phenomena that coexist in the brain. Segregation is the ability for specialized processing to occur within densely interconnected groups of areas, whereas integration is the ability to rapidly combine specialized information from distributed areas (Rubinov & Sporns, 2010). Complex networks present both high integration and segregation (Tononi, Sporns, & Edelman, 1994). The clustering coefficient is a measure of segregation, whereas global efficiency (Latora & Marchiori, 2001) is a measure of integration.

Since the network is weighted, the weighted version of the complex network measurements needs to be applied. Let *A* = {*a*_*mn*_} be the directed adjacency matrix (Albert & Barabási, 2002) of the network (*a*_*mn*_ = 1 when there is a connection from *m* to *n*, *a*_*mn*_ = 0 *otherwise*). Let also $d_{m}^{tot}$ be the total degree of node *m*, and $d_{m}^{↔}$ = ∑_*m*≠*n*_*a*_*mn*_*a*_*nm*_. The local clustering coefficient of node *m* for weighted networks is (Fagiolo, 2007):

$$C_{m}=\frac{{\left(\hat{W}+{\hat{W}}^{T}\right)}_{mm}^{3}}{2\left[d_{m}^{tot}\left(d_{m}^{tot}-1\right)-2d_{m}^{↔}\right]}$$ (9)

where $\hat{W}$ = *W*^1/3^, and ${\left(\hat{W}+{\hat{W}}^{T}\right)}_{mm}^{3}$ is the *m*th element of the main diagonal of ${\left(\hat{W}+{\hat{W}}^{T}\right)}^{3}$. Then, *C* = ∑_*m*_*C*_*m*_ is used as a measure of segregation.

The second measure we are going to compute is the global efficiency, calculated as (Latora & Marchiori, 2001; Rubinov & Sporns, 2010):

$$E=\frac{1}{N\left(N-1\right)}\sum_{i\neq j}{\left({\vec{l}}_{ij}\right)}^{-1}$$ (10)

where ${\vec{l}}_{ij}$ is the shortest weighted path length from *i* to *j*.

## RESULTS

### Global Complexity of Simulated Complex Networks

As stated before, the goal of this work is to propose a new measure of structural complexity that is useful for brain networks. To demonstrate the usefulness of the quantity we defined, we start by measuring how changes in the underlying network structure affect the observed values of global complexity. To this end, we devised a scenario in which the network gradually transforms from the perfectly orderly state (regular lattice network) to a completely random state (Erdos–Renyi network). Following Equations 7 and 8 we expect complexity to have a minimum at these states. Network states different from these minimums would have a mixture of order and disorder and thus were modeled using the small-world model (Watts & Strogatz, 1998). In this model, nodes of the network are placed on a regular *k*-dimensional grid and each node is connected to *m* of its nearest neighbors, producing a regular lattice of nodes with equal degrees. Then, with probability *p*, each connection is randomly rewired. The RL network corresponds to the value *p* = 0. When *p* > 0, edge rewiring is applied, and this changes the degree distribution of nodes. On the other end of the spectrum is the ER model (Erdös & Rényi, 1959), obtained when *p* = 1, in which there is no connectivity pattern between nodes. In between, small-world networks, obtained for values 0 < *p* < 1, present high clustering and short path length (Watts & Strogatz, 1998).

Graph theoretical studies of mammalian cortical networks recreated from tract tracing experiments demonstrated that the cat and macaque interareal anatomical networks share similar SW properties of short path length and high clustering (Hilgetag & Kaiser, 2004; Sporns & Zwi, 2004). Additionally, studies of anatomical and functional connectivity networks estimated from human neuroimaging data also found SW characteristics (Bassett & Bullmore, 2006; Salvador et al., 2005). To simulate RL, SW, and ER networks we use Matlab’s function WattsStrogatz.m, which has as inputs the parameters *k* and *p*.

**Figure 2.**
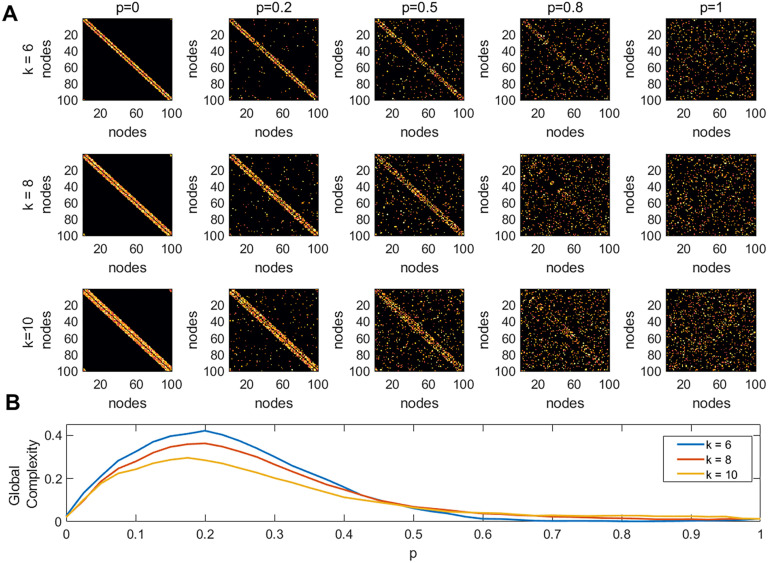
Global complexity of simulated networks. (A) All networks have the same size *N* = 100, and were simulated using the Watts and Strogatz algorithm for creating small-world networks. The inputs to the model are the rewiring probability *p*, and mean node degree k. (B) Global complexity as a function of the rewiring probability *p*.

Figure 2A shows examples of matrices of size *N* = 100, for five different values of the rewiring probability *p*, and three values of the mean node degree *k*. The weights in the network were generated from a uniform random distribution with values between 0 and 1. We then placed 10^4^ random walkers onto these networks. The steps for estimating the global complexity of the network are presented in Figure 1 and described in detail in the Methods section. Figure 2B shows the global complexity of a network as a function of the rewiring probability *p*. Three different values of the average node degree were used *k* = 6, 8, 10. The results show that for a fixed network size the maximum global complexity decreases with the increase of *k* (the network gets denser). Additionally, the probability at which the peak in complexity was achieved, also decreased with the increase of *k*.

### Complexity Analysis of Large-Scale Human Brain Networks

**Figure 3.**
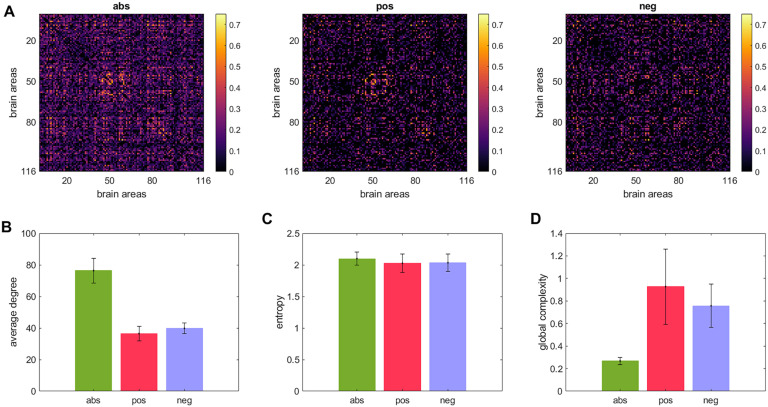
Global complexity of the entire brain network. (A) A matrix consisting of the absolute value of all connections (denoted as **abs**), a matrix consisting of only the positive connections (denoted as **pos**), and a matrix comprising the absolute value of only the negative connections (denoted as **neg**). (B) Node degree averaged across subjects. (C) Entropy averaged across subjects. (D) global complexity averaged across subjects.

Figure 3A displays the **abs**, **pos**, and **neg** matrices for one subject. Figure 3B shows the node degree of the three matrices average across all subjects, Figure 3C shows their entropy, and Figure 3D their global complexity. Our results show that the **pos** matrices are sparser than the **neg** matrices but have approximately the same entropy. This combination results in the **pos** network having a higher global complexity than the **neg** matrices. The **abs** matrices presented the lowest global complexity of the three cases. Note that the density of connections by itself is not a predictor of the global complexity of the network. If the raw fMRI data includes more positive correlations than negative, which is common, then we expect that removing the global signal increases negative correlations. This is verified by the increase in the density of negative correlations and the decrease in the density of positive correlations (Supporting Information Figure S2A) when removing the global signal. The average weights of the connections follow the same trend (Supporting Information Figure S2B). This seems to indicate that the spatial complexity of the network is linked to its density. However, our results indicate this is not the case. Supporting Information Table S1 shows the correlation between global complexity and network density for the **abs**, **pos**, and **neg** cases. As we can see, the only significant correlation (*r* = 0.59) was obtained for the **pos** case when removing the global signal; in all the other cases the correlations were small and nonsignificant.

**Figure 4.**
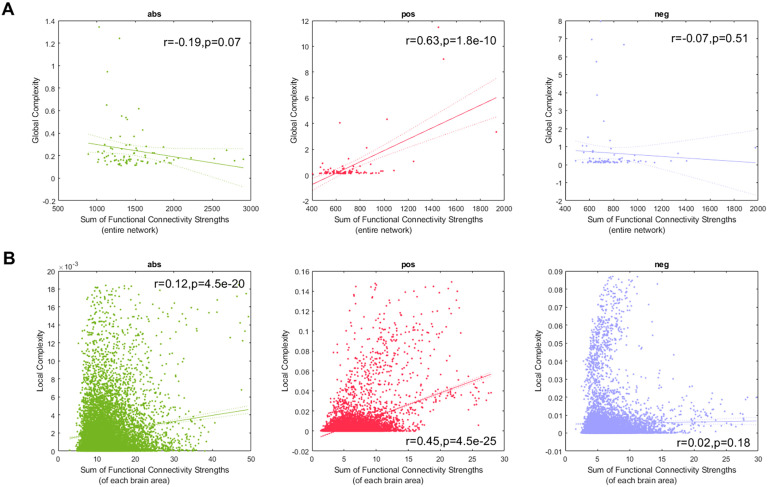
Link between complexity and connectivity. (A) Global complexity versus the sum of functional connectivity strengths for all subjects, resulting in a maximum of 89 points. (B) local complexities versus the sum of functional connectivity strengths, for all subjects and brain areas, resulting in a maximum of 89 × 116 = 10,324 points. The **abs**, **pos**, and **neg** networks appear in that order from left to right. In all panels, points outside of the percentiles 5 and 95 were classified as outliers and were removed.

Figure 4A shows the linear fits between the global complexity and the sum of the functional connectivity strengths (SFCS) of the entire brain network for the **abs**, **pos**, and **neg** cases. We found that for the **pos** case, there is a strong correlation (*r* = 0.63, *p* < 0.05) between global complexity and SFCS. The anticorrelation network (*r* = −0.07, *p* = 0.51) and the **abs** network (*r* = −0.19, *p* = 0.07) were not significantly correlated with SFCS. We also computed the linear fits between local complexities and the SFCS of each brain area (Figure 4B). We found that for the **pos** case the link between complexity and functional connectivity was significantly weaker at the local scale (*r* = 0.45, *p* < 0.05) compared with the global scale (Figure 4A). For the anticorrelation network there was no link at the local scale (*r* = 0.02, *p* = 0.18), while we found a weak correlation for the **abs** case (*r* = 0.12, *p* < 0.05).

In the analysis involving local complexities, the large number of samples (we consider all subjects and all brain areas resulting in a maximum of 89 × 116 = 10,324 points) allows us to compute the mutual information (MI) (Cover & Thomas, 2006) between local complexities and SFCS, which was not feasible for the analysis involving global complexities where we only had 89 data points. The advantage of using MI is that it is model free and can estimate nonlinear interactions, which are not possible to detect using the linear analysis presented in Figure 4. We computed MI with the MATLAB toolbox for the analysis of neuroscience data developed by Timme and Lapish (2018). A significant value was attached to the MI by using a surrogate data approach (Pereda, Quiroga, & Bhattacharya, 2005). We created surrogate data by randomly shuffling the local complexity series 1,000 times. For each new surrogate series, we computed its MI with the SFCS series. We then calculated the mean and the standard deviation of the MI surrogates and used the MI value obtained from the original signals to construct a Z-score. The results (Supporting Information Table S2) show that both **pos** and **neg** networks present a significant statistical dependence between local complexity and SFCS, being the dependence for the **pos** network more than 10 times stronger than the dependence for **neg** networks.

**Figure 5.**
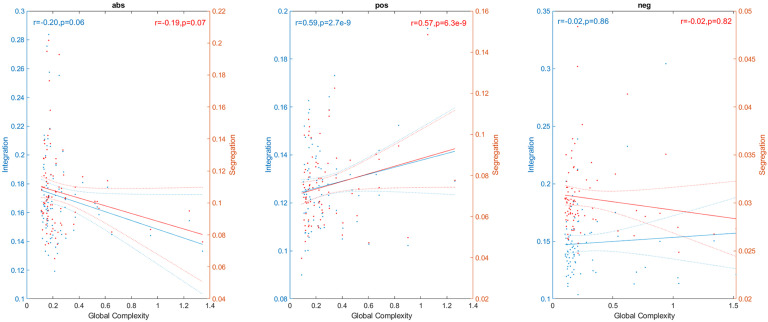
Link between global complexity and integration (blue) and segregation (red). Points outside of the percentiles 5 and 95 were classified as outliers and were removed.

Complex networks are expected to present high values of both integration and segregation. Thus, we also explored the link between them and global complexity (Figure 5). Integration and segregation were estimated using the global efficiency and average clustering coefficient of the network, respectively (Sporns, 2013). We found strong correlations between global complexity and both integration (*r* = 0.59, *p* < 0.05) and segregation (*r* = 0.57, *p* < 0.05) for the **pos** network, and no significant correlations for the **neg** case (*r* = −0.02, *p* = 0.86 for integration, and *r* = −0.02, *p* = 0.82 for segregation). Correlations were negative for the **abs** case (*r* = −0.20, *p* = 0.06 for the correlation with integration and *r* = −0.19, *p* = 0.07 for the correlation with segregation). Although nonsignificant, the *p* values were close to the *p* = 0.05threshold. These negative correlations are counterintuitive, since we expect that a complex network has both high values of integration and segregation, and as we increase the complexity of the network those topological values should also increase.

**Figure 6.**
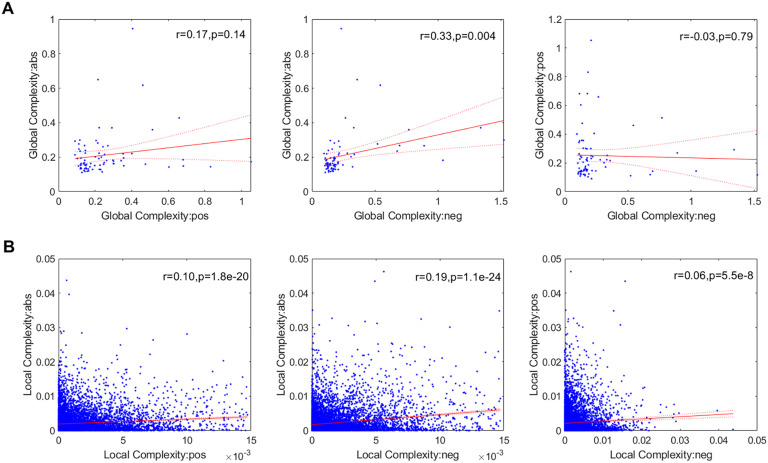
Link between the three network types (**abs**, **pos**, and **neg**) at the global (A) and local (B) scales. Points outside of the percentiles 5 and 95 were classified as outliers and were removed.

We also investigated the link between the three network types at the global (Figure 6A) and local scales (Figure 6B), finding that the **pos** and **neg** networks are not significantly correlated at any spatial scale.

**Figure 7.**
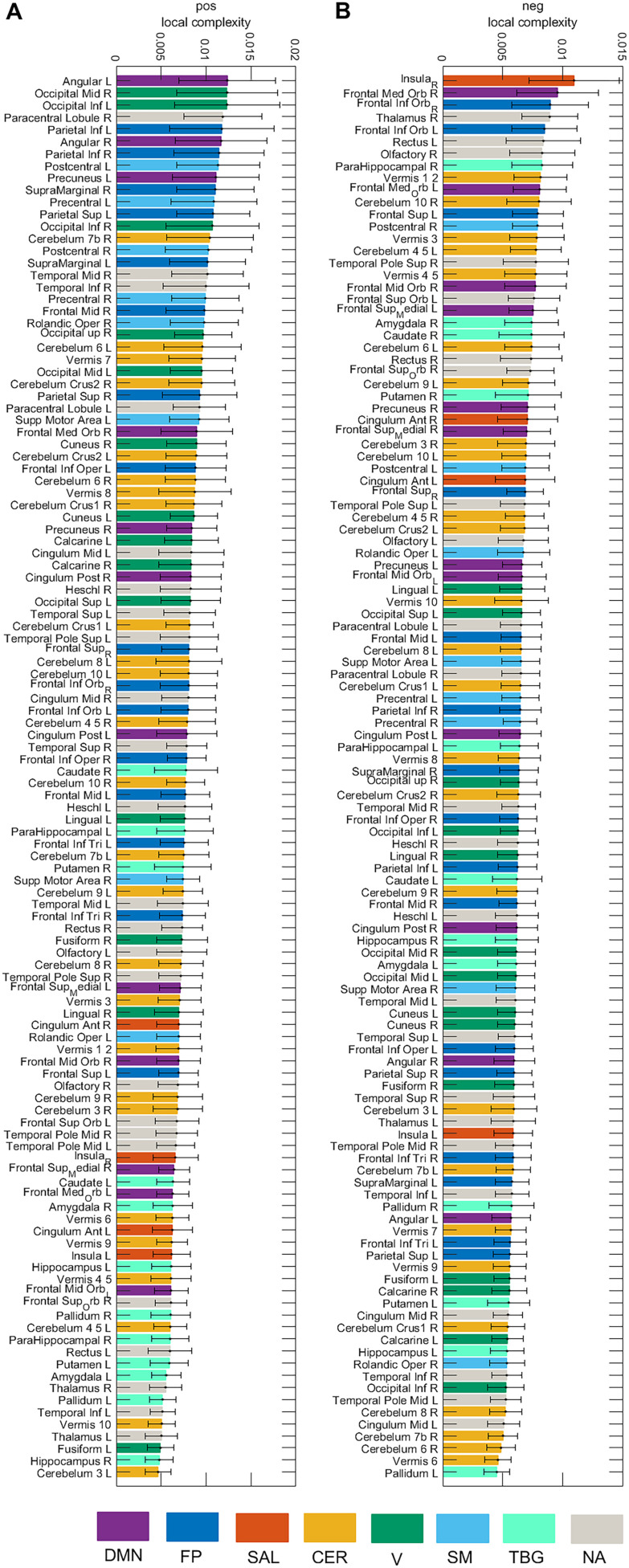
Local complexity of the 116 brain areas for the **pos** and **neg** cases. Seven resting-state networks (see Supporting Information Table S3) are represented through different colors: default mode network (DMN), fronto-parietal (FP), salience (SAL), sensorimotor (SM), visual (V), cerebellar (CER), and temporo-basal-ganglial (TBG) networks. The gray color represents areas not assigned (NA) to any of these networks.

**Figure 8.**
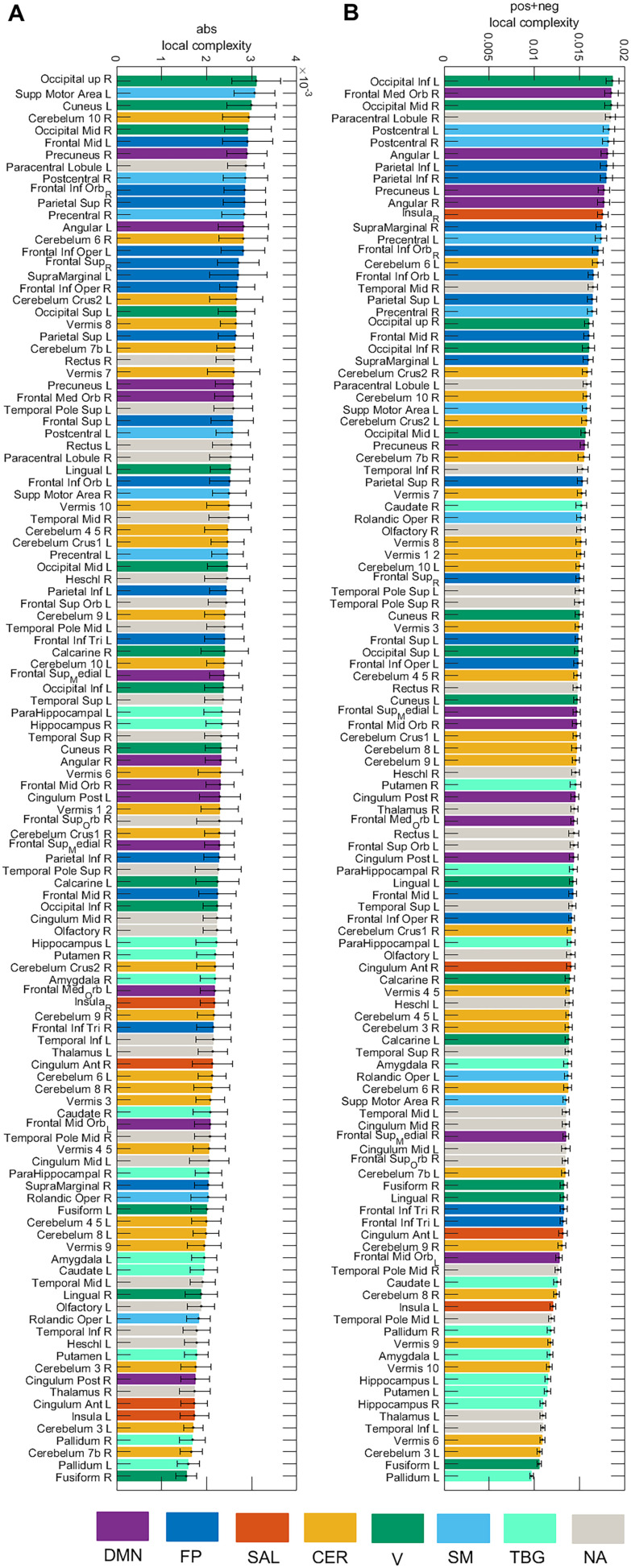
Local complexity of the 116 brain areas for the **abs** and **pos** + **neg** cases. Seven resting-state networks (see Supporting Information Table S3) are represented through different colors: default mode network (DMN), fronto-parietal (FP), salience (SAL), sensorimotor (SM), visual (V), cerebellar (CER), and temporo-basal-ganglial (TBG) networks. The gray color represents areas not assigned (NA) to any of these networks.

Figure 7 presents the local complexity of the 116 brain areas for the **pos** and **neg** cases. Seven resting-state networks (Sedeño et al., 2016) were considered (DMN, FP, SAL, CER, V, SM, TGB) as well as areas that were not allocated to a network (NA). In the **pos** case, the area with the highest complexity belongs to the DMN (Angular L), whereas for the **neg** case, the area belongs to the salience network (Insula R). Figure 8 displays the local complexity for the **abs** case and the sum of the complexities of the **neg** and **pos** case (**neg** + **pos**). In the **abs** case, the highest complexity was obtained for the Occipital_Sup_R, while the Occipital_Inf_L presented the highest complexity for the **neg** + **pos** case.

**Figure 9.**
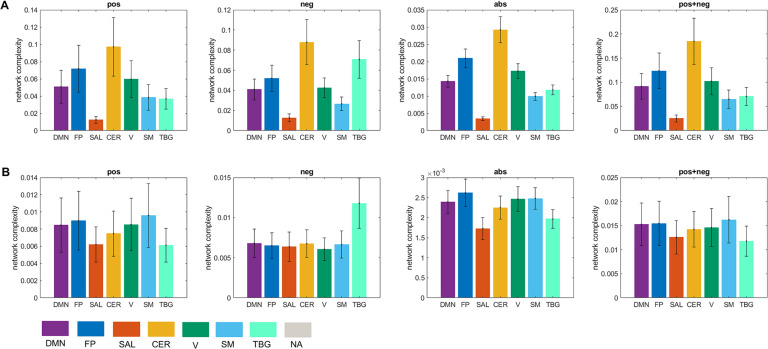
Network complexity. (A) Global complexity of seven resting-state networks. (B) Global complexity divided by the number of areas in each network. Seven resting-state networks (see Supporting Information Table S3) are represented through different colors: default mode network (DMN), fronto-parietal (FP), salience (SAL), sensorimotor (SM), visual (V), cerebellar (CER), and temporo-basal-ganglial (TBG) networks. The gray color represents areas not assigned (NA) to any of these networks.

We computed the global complexity of the seven resting-state networks (Figure 9A). We found that the network with the highest complexity for all cases was the cerebellar network, while the network with the lowest complexity was the salience network. The DMN, FP, CER, V, and SM networks presented more complexity in the **pos** than in the **neg** case, while the SAL and TGB networks were more complex in the **neg** case. When interpreting this result we need to be aware of the fact that since the global complexity of the network is computed as the sum of the local complexities (Equation 8), networks comprising few brain areas (as is the case of the salience network) will have a low value of global complexity provided that the difference in the values of the local complexities is not high (see Figures 7 and 8). To account for this issue, we also divided the global complexity of each network by the number of areas in each network (Figure 9B). As a result, although the average contribution of the areas in the salience network to the network complexity is still the lowest among the seven resting-state networks for the **pos** case, it is the areas in the visual network the ones with the lowest contribution in the **neg** case.

**Figure 10.**
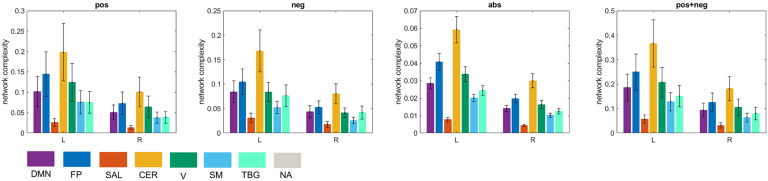
Interhemispheric asymmetry of global complexity. L = left hemisphere R = right hemisphere. Seven resting-state networks (see Supporting Information Table S3) are represented through different colors: default mode network (DMN), fronto-parietal (FP), salience (SAL), sensorimotor (SM), visual (V), cerebellar (CER), and temporo-basal-ganglial (TBG) networks. The gray color represents areas not assigned (NA) to any of these networks.

Along these lines, hemispherical differences can be investigated as well. Previous studies have found interhemispheric asymmetry in brain connectivity during resting state (Medvedev, 2014). We found that the left hemisphere was significantly more complex than the right hemisphere for the seven resting-state networks (Figure 10).

The results presented above were obtained in connectivity matrices obtained from fMRI signals where the global signal was removed. For comparison, we also computed the global and local complexities of the networks constructed from fMRI data without global signal regression. Supporting Information Figure S3 shows the local complexity of the 116 brain areas for the **pos** and **neg** cases, while Supporting Information Figure S4 shows the complexities for the **abs** and **pos** + **neg** cases. When comparing these results to the global signal regression case (Figures 7 and 8), we found that the rank of areas with high complexity changed completely. Additionally, we also computed the correlation between global complexity and the integration and segregation of the **abs**, **pos**, and **neg** networks. Our results show that while there is a strong correlation between these topological measures and global complexity when the global signal is regressed out (Figure 5) for the **pos** case, no significant correlation exists when the global signal is present (Supporting Information Figure S5).

Previously, we have focused on the complexity of fMRI-based brain networks. For comparison, we also analyzed the complexity of the anatomical network underlying brain activity. For this, the anatomical connectivity matrix of each subject was computed using the HCP preprocessed diffusion data (see Methods). Supporting Information Figure S6 shows the average anatomical connectivity across all subjects. Supporting Information Figure S7 shows the complexity of the 116 areas. Most of the top-ranked areas according to complexity values belong to the TBG network. Additionally, the global complexity of the anatomical matrix was not significantly correlated to the integration and segregation of the network (Supporting Information Figure S8).

## DISCUSSION

In this study, the complexity of each node in a network (i.e., local complexity) was computed using an index that compares the sample entropy of the time series generated by the movement of a random walker on the network, resulting from removing the node and its connections, to the sample entropy of the time series obtained from a regular lattice (the ordered state) and an Erdös–Rényi network (disordered state). Then, the network (global) complexity was constructed as the sum of the complexities of its nodes. Our simulations demonstrated that our measure of complexity (Equations 7 and 8), achieves a minimum for the regular lattice and Erdös–Rényi networks, and a maximum at some intermediate state, representing a small-world network with both order and disorder characteristics (Figure 2).

The rationale behind the use of random walks is that diffusion processes are capable of uncovering the large-scale topological structure of complex networks (Noh & Rieger, 2004; Simonsen, Astrup Eriksen, Maslov, & Sneppen, 2004; Skardal & Adhikari, 2018). For instance, random walks are the basis of Infomap (Rosvall & Bergstrom, 2008), a popular method for detecting community structure in complex networks. Past studies of anatomical and functional brain connectivity have found interlinked communities that form a partly decomposable modular architecture (Ashourvan, Telesford, Verstynen, Vettel, & Bassett, 2019; Meunier, Lambiotte, Fornito, Ersche, & Bullmore, 2009). Such architectures are hallmarks of complex systems and are thought to be of fundamental importance for understanding mental processing and cognition (Bola & Borchardt, 2016). In the brain, hierarchies of linked communities span several levels including brain regions, functional circuits, and large-scale networks. This structural diversity cannot be captured by previous structural complexity measures relying mainly on Shannon entropy (Shannon, 1948), but can be probed using random walks (Rosvall & Bergstrom, 2008).

Once we constructed the time series of the random walker’s movement in the network, we needed a measure to estimate its complexity. There is a diversity of complexity measures based on different entropy definitions, such as Shannon entropy (Shannon, 1948), Tsallis entropy (Tsallis, 1988), spectral entropy (Inouye et al., 1991), wavelet entropy (Rosso et al., 2001), approximate entropy (Pincus, 1991), sample entropy (Richman & Moorman, 2000), fuzzy entropy (Chen, Wang, Xie, & Yu, 2007), and permutation entropy (Bandt & Pompe, 2002).

In this work we selected sample entropy as it quantifies the amount of regularity and unpredictability of fluctuations in a time series (Richman & Moorman, 2000). This is important because of the presence of communities in brain networks (Ashourvan et al., 2019; Meunier et al., 2009), which will result in repetitive patterns of nodes in the time series of the random walker’s movement (Fortunato & Hric, 2016; Sanchez-Rodriguez, Iturria-Medina, Mouches, & Sotero, 2019). On the other hand, this same community structure will result in a random walker’s movement that can be decomposed into different oscillatory modes (or temporal scales). This is because the random walker will spend considerable time in large communities (reflected in slow modes) and significantly less time in smaller clusters (reflected in fast modes). These multiple temporal scales are not considered in the sample entropy method, which is based on a single temporal scale. To address this issue in future works, we propose to use the multiscale entropy method (Costa, Goldberger, & Peng, 2005), which computes sample entropy at shorter and longer timescales, and the quantification of the overall entropy of the time series is computed as the sum of the entropy values over all individual timescales.

Our study of brain complexity found interhemispheric asymmetry, where the left hemisphere was significantly more complex than the right hemisphere, for all the seven brain networks explored. Previous studies have also found interhemispheric asymmetry in brain connectivity during resting state. For instance, a recent study used near-infrared spectroscopy signals to estimated functional connectivity matrices (Medvedev, 2014). Their results revealed significantly stronger and denser connectivity patterns in the right hemisphere in most subjects. This denser pattern of connections in the right hemisphere compared with the left hemisphere can lead to a lower structural complexity if it is not accompanied with a significant increase in the entropy of the network. Thus, the balance between the entropy of the network and its density determines the network’s complexity. This was exemplified in Figure 3 where we found that the entropy of the positive network and the anticorrelated network were essentially the same, but the positive network was sparser, which resulted in it being more complex than the anticorrelated network.

Finally, we found that the complexity of the **pos** network is correlated to functional connectivity between the brain areas comprising the network, as well as to the integration and segregation of the network, suggesting the **pos** network is related to the information processing in the brain. On the other hand, the **neg** network presented a weaker (although statistically significant) nonlinear dependence between local complexity and functional connectivity than the **pos** network. Although weaker than the **pos** case, this dependence should not be neglected. For example, a recent study has shown that the inclusion of anticorrelations improved the performance of a support vector machine model for classifying autism spectrum disorder by using fMRI-based functional connectivity data (Kazeminejad & Sotero, 2019). Based on these results, we suggest that functional connectivity studies should analyze **pos** and **neg** networks separately, instead of the **abs** network as is commonly done.

## ACKNOWLEDGMENTS

Data were provided, in part, by the Human Connectome Project, WU-Minn Consortium (Principal Investigators: David Van Essen and Kamil Ugurbil; 1U54MH091657), funded by the 16 NIH Institutes and Centers that support the NIH Blueprint for Neuroscience Research, and by the McDonnell Center for Systems Neuroscience at Washington University.

## SUPPORTING INFORMATION

Supporting Information for this article is available at https://doi.org/10.1162/netn_a_00138.

## AUTHOR CONTRIBUTIONS

Roberto Sotero: Conceptualization; Formal analysis; Funding acquisition; Investigation; Methodology; Project administration; Resources; Software; Supervision; Validation; Visualization; Writing - Original Draft. Lazaro M. Sanchez-Rodriguez: Conceptualization; Methodology; Software; Writing - Original Draft. Narges Moradi: Data curation; Software; Writing - Original Draft. Mehdy Dousty: Data curation; Software; Writing - Original Draft.

## FUNDING INFORMATION

Roberto Sotero, Canadian Network for Research and Innovation in Machining Technology, Natural Sciences and Engineering Research Council of Canada (http://dx.doi.org/10.13039/501100002790), Award ID: RGPIN-2015-05966.

## TECHNICAL TERMS

Random Walk: A succession of random steps along some mathematical space.
Functional connectivity: Statistical dependencies between the time series of different brain areas.
Global signal: The mean time course computed over all voxels within the brain. It is used a normalization factor for removing the effects of global variations in fMRI data.
Pearson correlation: A number between − 1 and 1 that indicates the strength of the linear relationship between two variables.
Functional magnetic resonance imaging (fMRI): Detect changes in blood oxygenation and flow that occur in response to neuronal activity.
Network integration: The combination of information exclusive to specialized brain regions.
Network segregation: Processes occur separately in groups of interconnected populations or regions.
Clustering coefficient: Ratio between existing and possible number of triangle motifs in a network. Measure of network segregation.
Global efficiency: Measures how efficiently the information is exchanged in the network. Measure of network integration.
Erdos–Renyi network: A random graph where each possible edge has the same probability of existing.
Small-world network: Network with high clustering coefficient and short average path length.
Mutual information: Quantifies the reduction in uncertainty about one random variable through observing another variable.
Anatomical connectivity: Network of structural (synaptic) connections linking sets of neurons, neuronal populations, or brain areas.
