# Supplementary material for: Estimation of global and local complexities of brain networks: A random walks approach
Source: Netw Neurosci. 2020 Jul 1;4(3):575–94. doi: 10.1162/netn_a_00138 (PMC7462425; doi:10.1162/netn_a_00138)
Supplement: Supplementary file 1 [file netn-04-575-s001.pdf]

Sotero, R. C., Sanchez-Rodriguez, L. M., Moradi, N., Dousty, M. (2020). Supporting information for "Estimation of Global and Local Complexities of Brain Networks: A Random Walks Approach." *Network Neuroscience*, *4*(3), 575–594. https://doi.org/10.1162/netn_a_00138

Table S1. Correlation between global complexity and the density of connections in the brain network. Two cases were considered: the global signal was removed (gsr), the raw signal was used (no gsr). Significant correlations ($p<0.05$) are highlighted in yellow.

	**Corr (density,global complexity)**		
	**abs**	**pos**	**neg**
**no gsr**	0.17	0.03	-0.16
**gsr**	0.08	0.59	-0.07

Table S2. Mutual Information between local complexity and the sum of functional connectivity strengths (for all subjects and brain areas, resulting in a maximum of 89x116=10324 points). Z-scores are significant for $|Z| > 1.96$.

**Network**	**MI between local complexity and functional connectivity**	**Z-score**
abs	0.0083 bits	26.91
pos	0.0561 bits	209.51
neg	0.0040 bits	12.24

Table S3. Resting-state networks

Network	Areas
Default mode network (DMN)	Frontal_Mid_Orb_L, Frontal_Mid_Orb_R<br>Frontal_Sup_Medial_L, Frontal_Sup_Medial_R<br>Frontal_Med_Orb_L , Frontal_Med_Orb_R<br>Cingulum_Post_L, Cingulum_Post_R<br>Angular_L, Angular_R<br>Precuneus_L, Precuneus_R
Frontoparietal (FP)	Frontal_Sup_L, Frontal_Sup_R<br>Frontal_Mid_L, Frontal_Mid_R<br>Frontal_Inf_Oper_L, Frontal_Inf_Oper_R<br>Frontal_Inf_Tri_L, Frontal_Inf_Tri_R<br>Frontal_Inf_Orb_L, Frontal_Inf_Orb_R<br>Parietal_Sup_L, Parietal_Sup_R<br>Parietal_Inf_L, Parietal_Inf_R<br>SupraMarginal_L, SupraMarginal_R
Salience (SAL)	Insula_L, Insula_R<br>Cingulum_Ant_L, Cingulum_Ant_R
Sensorimotor (SM)	Precentral_L, Precentral_R<br>Rolandic_Oper_L, Rolandic_Oper_R<br>Postcentral_L, Postcentral_R<br>Supp_Motor_Area_L, Supp_Motor_Area_R
Visual (V)	Calcarine_L, Calcarine_R<br>Cuneus_L, Cuneus_R<br>Lingual_L, Lingual_R<br>Occipital_Sup_L, Occipital_Sup_R<br>Occipital_Mid_L, Occipital_Mid_R<br>Occipital_Inf_L, Occipital_Inf_R<br>Fusiform_L, Fusiform_R
Cerebellar (CER)	Cerebelum_Crus1_L, Cerebelum_Crus1_R<br>Cerebelum_Crus2_L, Cerebelum_Crus2_R<br>Cerebelum_3_L, Cerebelum_3_R<br>Cerebelum_4_5_L, Cerebelum_4_5_R<br>Cerebelum_6_L, Cerebelum_6_R<br>Cerebelum_7b_L, Cerebelum_7b_R<br>Cerebelum_8_L, Cerebelum_8_R<br>Cerebelum_9_L, Cerebelum_9_R<br>Cerebelum_10_L, Cerebelum_10_R<br>Vermis_1_2, Vermis_3, Vermis_4_5, Vermis_6<br>Vermis_7, Vermis_8, Vermis_9, Vermis_10
Temporo-basal-ganglial (TBG)	Hippocampus_L, Hippocampus_R<br>ParaHippocampal_L, ParaHippocampal R<br>Amygdala_L, Amygdala_R,<br>Caudate_L, Caudate_R<br>Putamen_L, Putamen_R<br>Pallidum_L, Pallidum_R

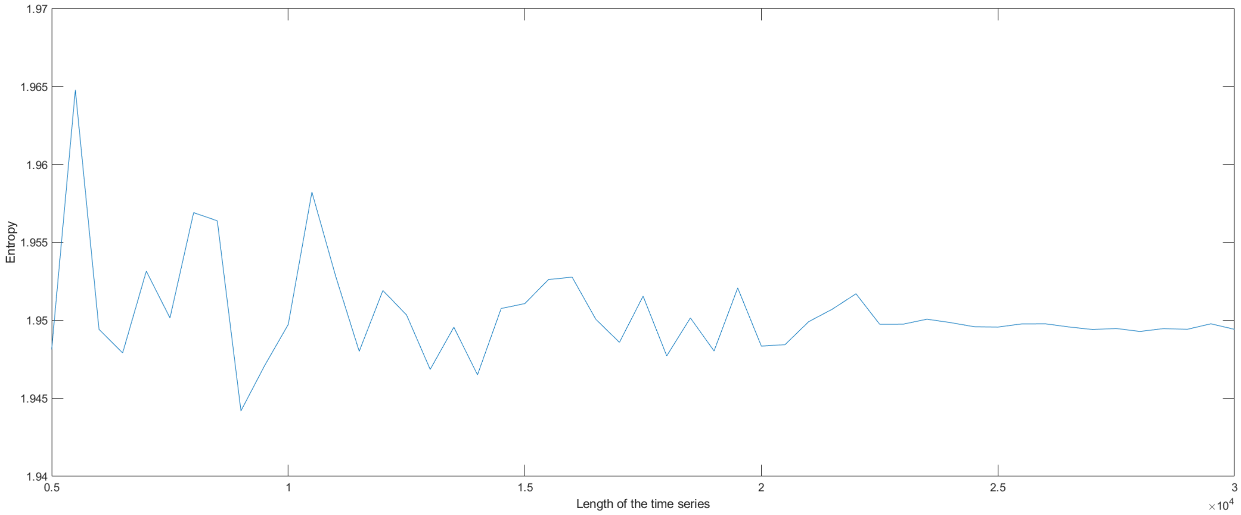

Figure S1. Sample entropy for different lengths of the time series. The series were constructed with the strengths of the nodes visited by a random walker released on an ER network (N=100).

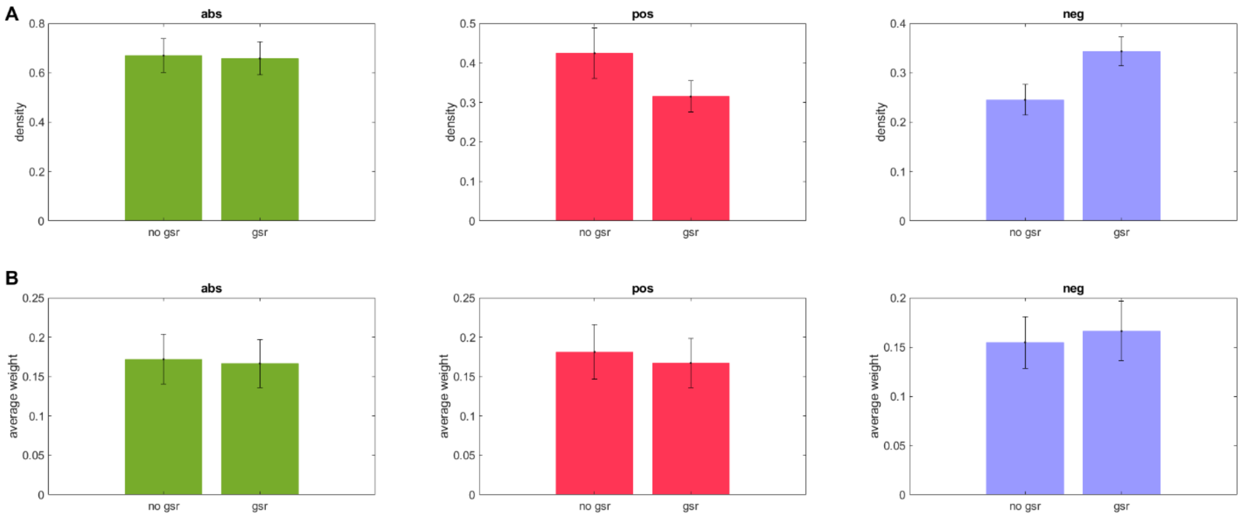

Figure S2. Density and average weight of the connections of the three (abs, pos, and neg) networks for the cases when the global signal was removed (gsr) and when the raw signal was used (no gsr).

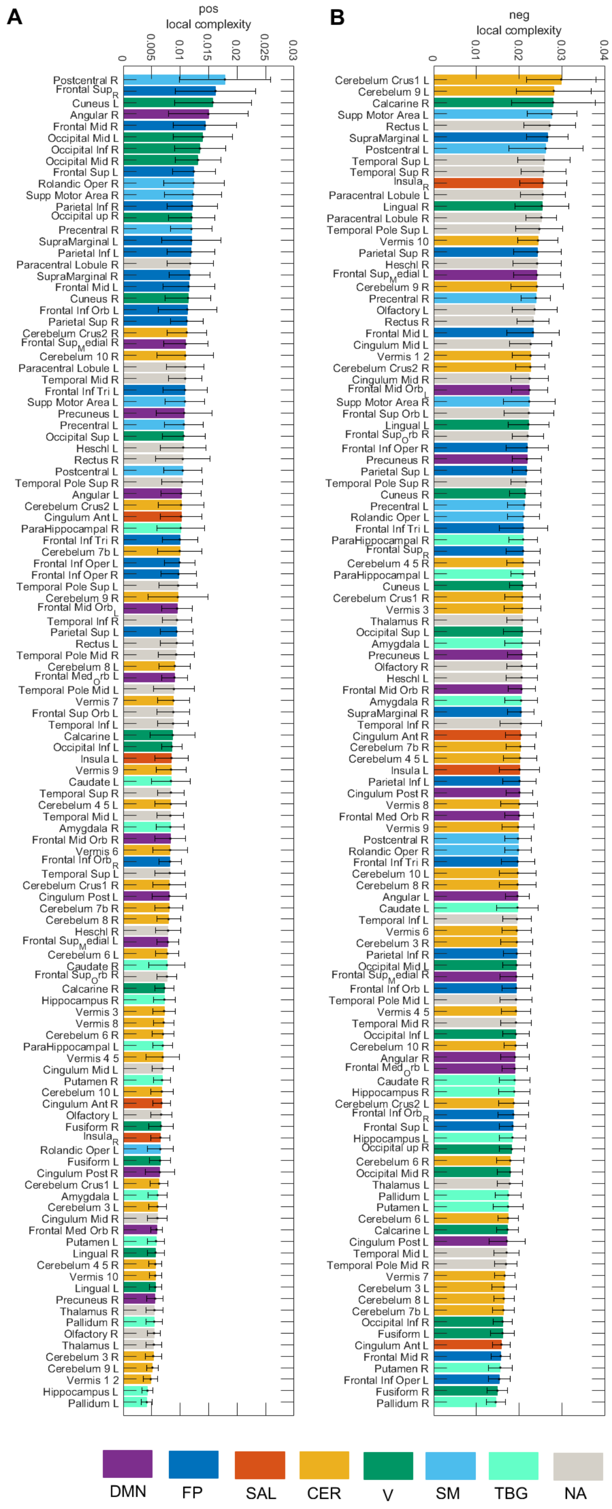

Figure S3. Local complexity of the 116 brain areas in the **pos** and **neg** networks for the case when the global signal was not removed. Seven resting-state networks (see Table S1) are represented through different colors: default mode network (DMN), frontoparietal (FP), salience (SAL), sensorimotor (SM), visual (V), cerebellar (CER), and temporo-basal-ganglial (TBG) networks. The gray color represents areas not assigned (NA) to any network.

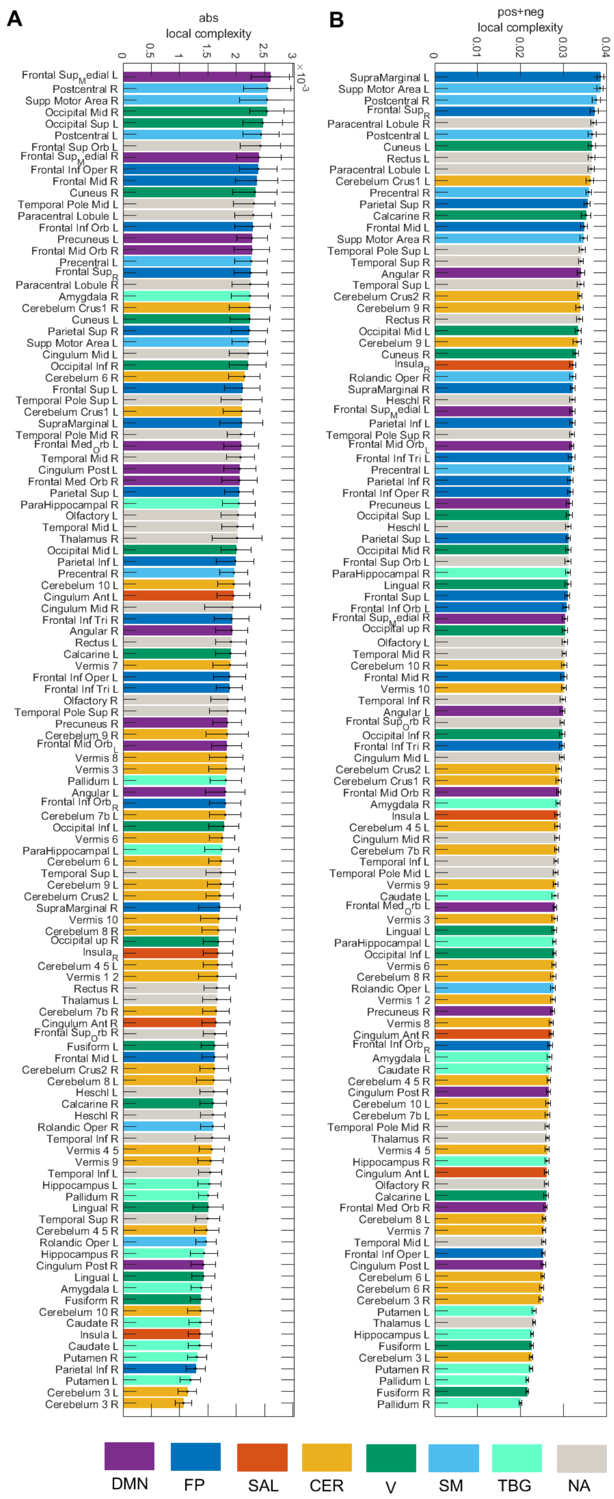

Figure S4. Local complexity of the 116 brain areas in the **abs** and **pos+neg** networks for the case when the global signal was not removed. Seven resting-state networks (see Supplementary Table 1) are represented through different colors: default mode network (DMN), frontoparietal (FP), salience (SAL), sensorimotor (SM), visual (V), cerebellar (CER), and temporo-basal-ganglial (TBG) networks. The gray color represents areas not assigned (NA) to any network.

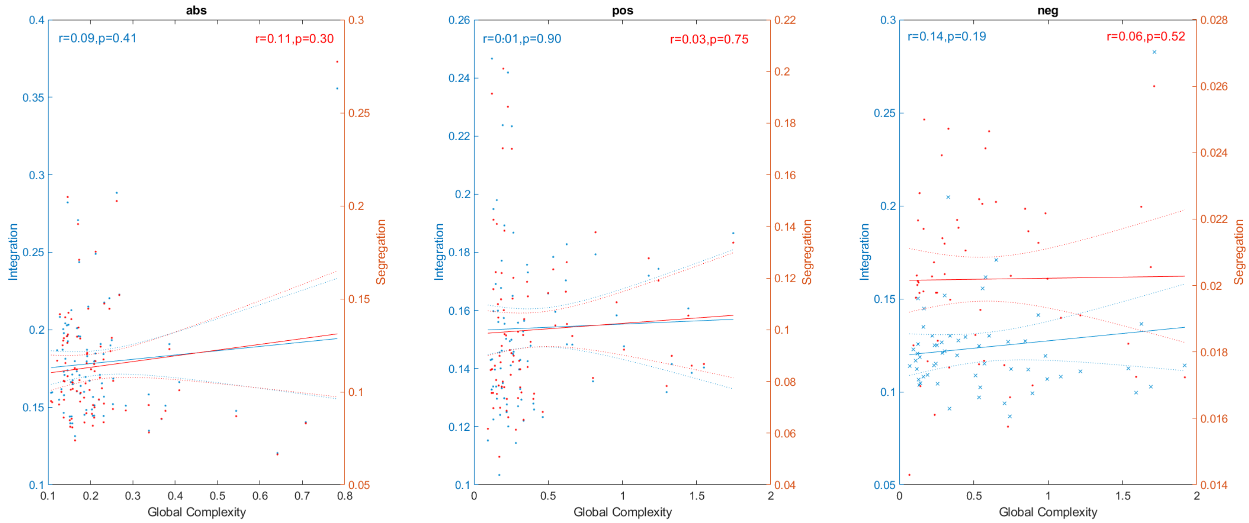

Figure S5. Relationship between global complexity and integration (blue) and segregation (red) for the case when the global signal was not removed. Points outside of the percentiles 5 and 95 were classified as outliers and removed.

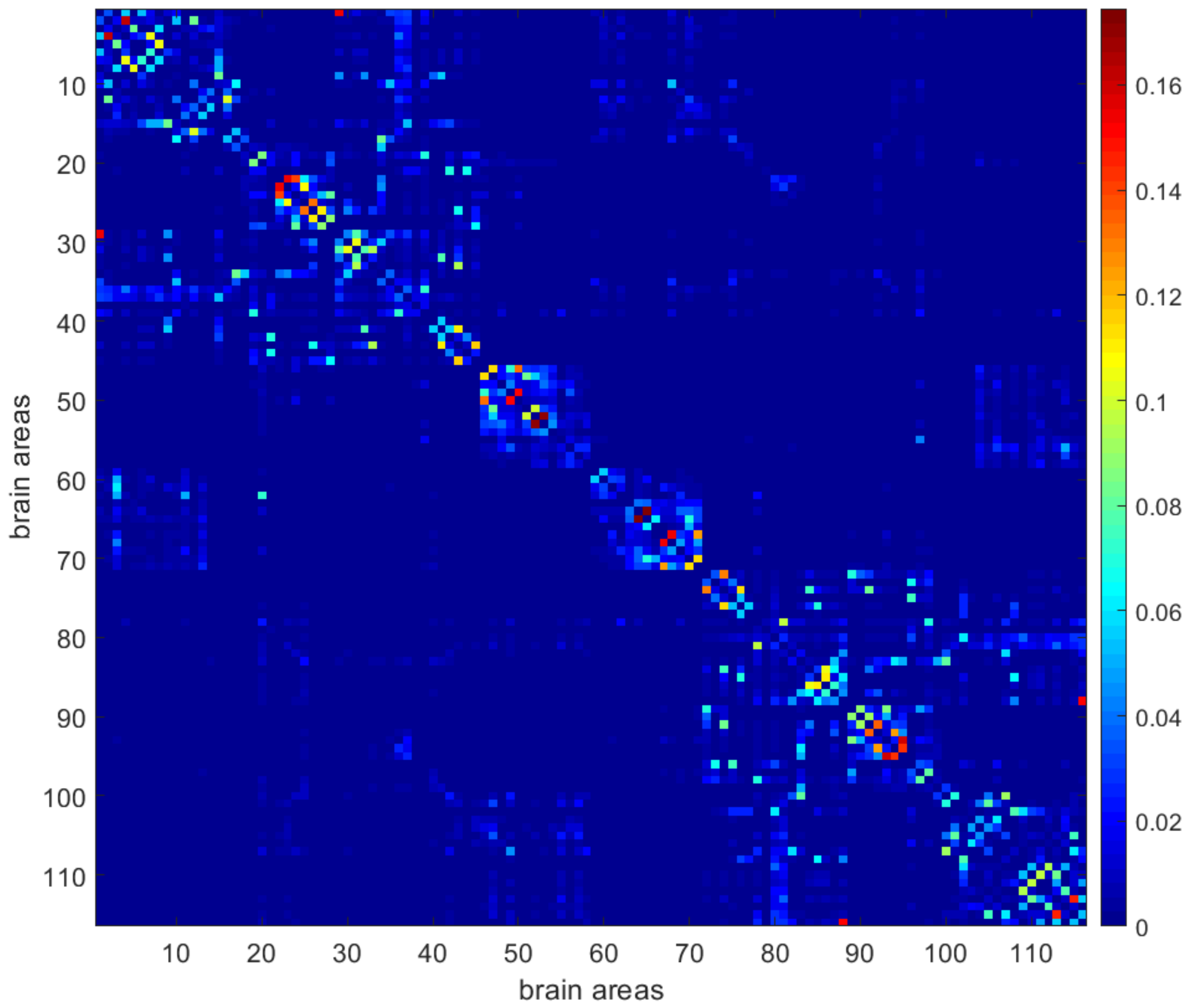

Figure S6. Average anatomical connectivity across all subjects (see section 2.3).

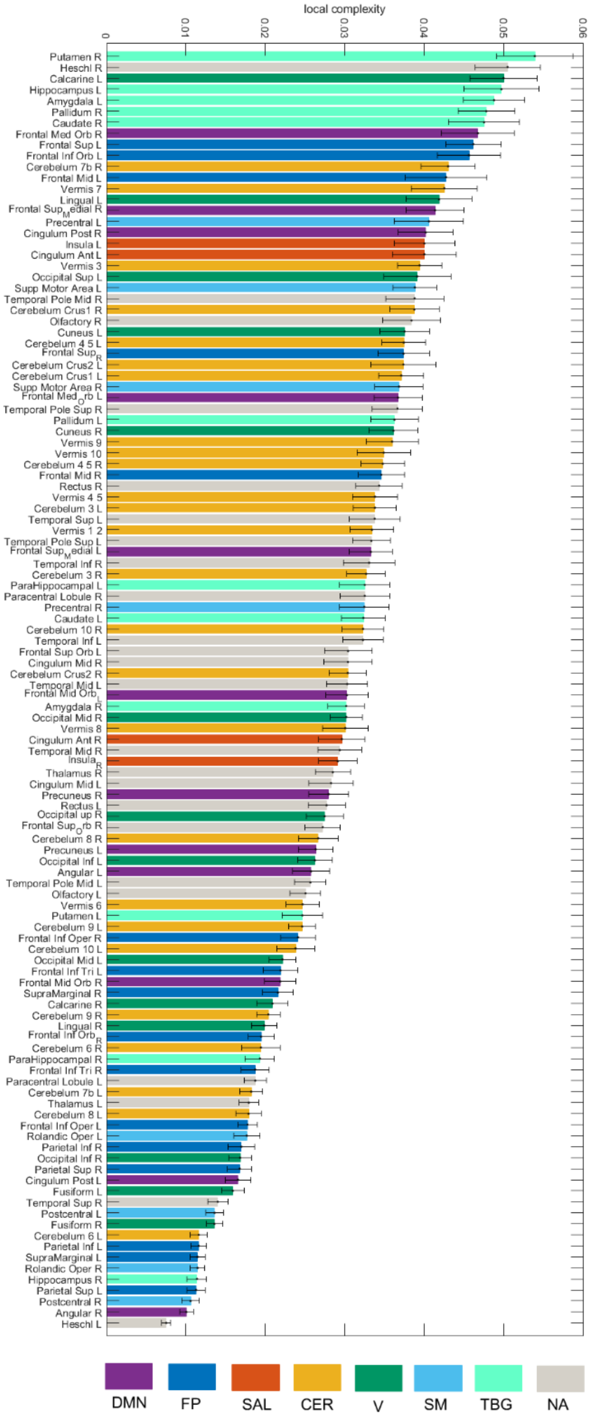

Figure S7. Local complexity of the 116 brain areas when using the anatomical connectivity matrix (section 2.3). For comparison to previous results, seven resting-state networks (see Table S1) are represented through different colors: default mode network (DMN), frontoparietal (FP), salience (SAL), sensorimotor (SM), visual (V), cerebellar (CER), and temporo-basal-ganglial (TBG) networks. The gray color represents areas not assigned (NA) to any network.

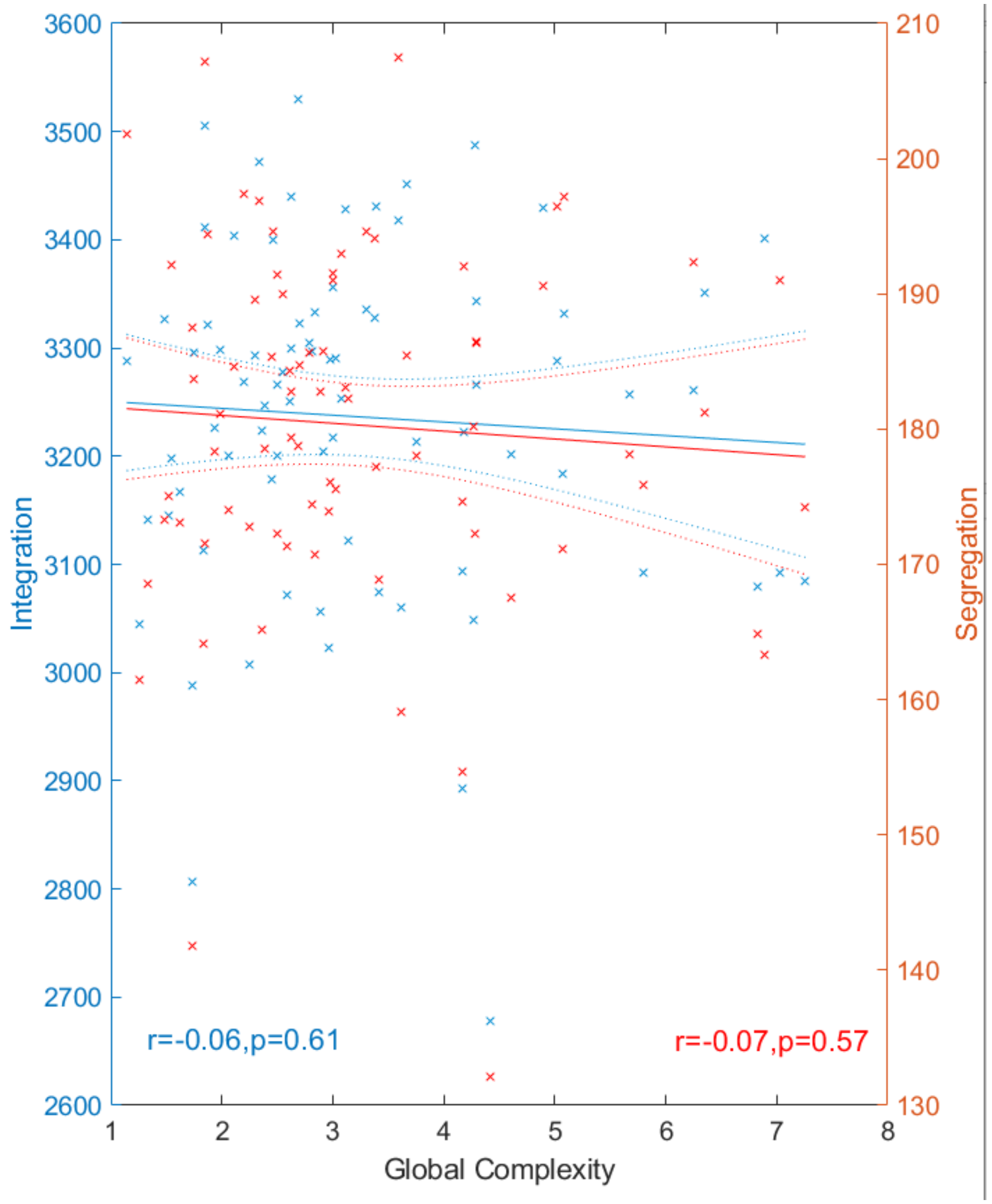

Figure S8. Relationship between global complexity and integration (blue) and segregation (red) for the case of the anatomical connectivity matrix (section 2.3). Points outside of the [ercenti;es 5 and 95 were classified as outliers and were removed.
